# Patient Reported Outcome Measures in Adults with Fontan Circulatory Failure

**DOI:** 10.3390/jcm13144175

**Published:** 2024-07-17

**Authors:** Guillermo Agorrody, Isaac Begun, Subodh Verma, C. David Mazer, Maria Luz Garagiola, Beatriz Fernandez-Campos, Ronald Acuña, Katherine Kearney, Alvan Buckley, Nitish K. Dhingra, Ehsan Ghamarian, S. Lucy Roche, Rafael Alonso-Gonzalez, Rachel M. Wald

**Affiliations:** 1 Toronto Adult Congenital Heart Disease Program, Peter Munk Cardiac Centre, Division of Cardiology, University Health Network, University of Toronto, Toronto, ON M5G 2N2, Canada; 2 Division of Cardiac Surgery, Unity Health, St. Michael’s Hospital, University of Toronto, 585 University Avenue, Toronto, ON M5G 2N2, Canadanitish.dhingra@mail.utoronto.ca (N.K.D.); 3 Department of Anaesthesia, Unity Health, St. Michael’s Hospital, University of Toronto, Toronto, ON M5S 1A1, Canada; 4 Applied Health Research Centre, Unity Health, St. Michael’s Hospital, University of Toronto, Toronto, ON M5S 1A1, Canada

**Keywords:** patient reported outcomes, Kansas City Cardiomyopathy Questionnaire, adult congenital heart disease, Fontan procedure, Fontan circulatory failure, heart failure

## Abstract

**Background:** Patient reported outcomes (PROs) are important measures in acquired heart disease but have not been well defined in Adult Congenital Heart Disease (ACHD). Our aim was to explore the discriminatory capacity of PRO survey tools in Fontan circulatory failure (FCF). **Methods:** Consecutive adults were enrolled from our ambulatory clinics. Inclusion criteria were age ≥18 years, a Fontan circulation or a hemodynamically insignificant shunt lesion, and sufficient cognitive/language abilities to complete PROs. A comprehensive package of PRO measures, designed to assess perceived health-related quality of life (HRQOL) was administered (including the Kansas City Cardiomyopathy Questionnaire [KCCQ-12], EuroQol-5-dimension [EQ5D], Short Form Health Status Survey [SF-12], self-reported New York Heart Association [NYHA] Functional Class, and Specific Activity Scale [SAS]). **Results:** We compared 54 Fontan patients (35 ± 10 years) to 25 simple shunt lesion patients (34 ± 11 years). The KCCQ-12 score was lower in Fontan versus shunt lesion patients (87 [IQR 79, 95] versus 100 [IQR 97, 100], *p*-value < 0.001). The FCF subgroup was associated with lower KCCQ-12 scores as compared with the non-FCF subgroup (82 [IQR 56, 89] versus 93 [IQR 81, 98], *p*-value = 0.002). Although the KCCQ-12 had the best discriminatory capacity for determination of FCF of all PRO tools studied (c-statistic 0.75 [CI 0.62, 0.88]), superior FCF discrimination was achieved when the KCCQ-12 was combined with all PRO tools (c-statistic 0.82 [CI 0.71, 0.93]). **Conclusions:** The KCCQ-12 questionnaire demonstrated good discriminatory capacity for the identification of FCF, which was further improved through the addition of complementary PRO tools. Further research will establish the value of PRO tools to guide management strategies in ACHD.

## 1. Introduction

Of all congenital defects diagnosed in the newborn, congenital heart disease (CHD) is the most common, with a global birth prevalence of up to 1% [1]. The population of adult survivors with complex CHD represents a unique and expanding cohort reflecting progressive improvements in medical, interventional, and surgical care [2]. Patients with congenital heart defects that are incompatible with a biventricular circulation represent a particularly vulnerable cohort. In such cases, a series of palliative operations designed to ultimately achieve a Fontan circuit are performed. The Fontan circulation is created to redirect systemic venous return into the pulmonary circulation without an intervening subpulmonary ventricle. This is achieved by direct connection of the superior and inferior vena caval systems to the pulmonary arteries. The classic Fontan palliation surgery included a direct connection of the right atrium to the pulmonary artery, whereas contemporary techniques incorporate an extracardiac conduit or intra-atrial lateral tunnel to connect the inferior vena cava to the pulmonary artery, while the superior vena cava is anastomosed separately to the pulmonary artery (typically in a staged approach).

Despite significant improvements in short-term technical success and survival in childhood, there is an escalating burden of long-term morbidity and mortality among Fontan patients arising from intersecting cardiac and extracardiac complications. Fontan circulatory failure (FCF), affecting more than 40% of adult Fontan patients, is the invariable end-stage consequence of these complications and is the leading cause of premature mortality in this population [3,4].

Adverse cardiovascular outcomes have traditionally incorporated clinical events such as mortality, arrhythmia, stroke, and heart failure (HF). More recently, patient reported outcome (PRO) measures have emerged as relevant metrics for cardiac patients with acquired heart disease and are now included as independent outcome measures in contemporary cardiovascular trials [5]. For example, the Kansas City Cardiomyopathy Questionnaire (KCCQ) is considered a validated survey tool, which was created with input from patients and clinicians, to capture domains which specifically affect the lives of patients suffering from acquired HF [6,7]. It is noteworthy that the KCCQ is currently recognized by the United States Food and Drug Administration as an accepted clinical outcome measure, suggesting that future approval of medical therapies may be contingent on the demonstration of clinical benefit with respect to KCCQ measures.

The incorporation of PROs as an endpoint in clinical investigations represents an important paradigm shift for patients with HF in the context of biventricular circulation. However, there is a paucity of data with respect to the role of PROs in therapeutic studies for patients with CHD, with scarce data available in the adult Fontan population. While some have advocated for the broad creation of Adult Congenital Heart Disease (ACHD) PRO metrics to assess quality of life (QOL) in the wider ACHD population; drawbacks to this approach include the heterogeneity of anatomies and the spectrum of physiologies under the umbrella of ACHD, some of which may not be equally suited to all PRO tools [8]. Indeed, the identification of reliable, reproducible, and suitable PRO measures is a growing concern for ACHD providers.

In this study, our specific aims were therefore to (1) assess the performance of a variety of PRO tools for the characterization of ambulatory Fontan patients, and to (2) characterize the discriminatory capacity of each PRO tool for the identification of FCF in the ambulatory adult Fontan population. We hypothesized that the KCCQ-12 questionnaire, a validated HF-specific health-related quality of life (HRQOL) survey tool, would demonstrate the highest discriminatory capacity for the identification of FCF.

## 2. Methods

### 2.1. Study Population

Consecutive adults with a Fontan circulation were approached for enrollment from the Toronto ACHD Program ambulatory clinic at the Toronto General Hospital in the University Health Network (September–December 2023). For purposes of comparison, consecutive adults with a simple shunt lesion, such as small atrial or ventricular septal defects or a small patent ductus arteriosus, were also enrolled from the same ambulatory clinics during the same time frame. Specific study inclusion criteria were (1) age ≥18 years; (2) CHD consisting of either a history of a Fontan palliation or a hemodynamically insignificant shunt lesion; and (3) sufficient cognitive and language abilities to complete PRO survey tools. Exclusion criteria consisted of the inability to complete at least 1 PRO survey tool. For the simple shunt lesion population, hemodynamically insignificant shunt lesions were defined as those restrictive to pressure and volume loading (i.e., normal right heart chamber dimensions in patients with pre-tricuspid shunts and normal left ventricular size with a pressure gradient >4 m/s for those with post-tricuspid shunts). In the Fontan population, those with FCF were approached at designated ACHD-HF clinics having individually met the international consensus definition of FCF as previously described and as subsequently verified by the study investigators [9]. At the time of study enrollment, clinical data were abstracted from the electronic health record (including demographics, cardiac anatomy, previous surgical and catheter interventional history, and results of routine clinical testing [i.e., electrocardiograms, echocardiograms, cardiopulmonary exercise testing, etc.]). It is our institutional practice to have all Fontan patients complete comprehensive bloodwork during ambulatory clinic visits, along with regular echocardiography and intermittent cardiopulmonary exercise testing. For the purposes of this study, we recorded clinical data at or close to the time of enrollment. All data were entered into a secure database in a deidentified format. Complementary HRQOL survey tools were completed (as detailed below).

### 2.2. PRO Survey Tools

A comprehensive package of PRO survey tools, selected to illustrate the multi-faceted characteristics of HRQOL in ACHD, was presented to each participant for completion at a single sitting at the time of their ambulatory clinic visit (shown in Table 1) [10]. We focused on defining perceived health status, as determined by HRQOL metrics, to capture physical health and/or functional status [11]. In total, we selected five HRQOL questionnaires based on previous reports in individuals with CHD and/or HF [6,7,11,12,13,14]. The specific survey tools used were (1) KCCQ-12; (2) EuroQol-5 Dimension (EQ-5D) 3 level version (3L) with Visual Analogue Scale (VAS); (3) Short Form Health Status Survey [SF-12v2]; (4) self-reported New York Heart Association (NYHA) class; and (5) Specific Activity Scale [SAS] class (Table 1).

### 2.3. Statistical Analysis

Categorical data are reported using counts and percentages. We assessed the normality of continuous variables using the Shapiro–Wilk test. We used mean and standard deviation (SD) for normally distributed data and median and interquartile range (IQR) for skewed data. Kruskal–Wallis and chi-square tests were used to conduct univariate comparisons between sociodemographic and medical variables of interest for continuous and categorical PRO measures, respectively. The approximated Wilcoxon rank sum test was used to determine the difference between the two groups according to baseline PROs. The c-statistic was used to evaluate the discriminatory capacity of a survey tool to identify FCF within the Fontan group. To evaluate the performance of the PROs to predict the binary outcome (class [FCF+ or FCF−]), we extracted predicted probabilities from a logistic regression model. We assessed model discriminative ability using Receiver Operating Characteristic (ROC) curve analysis, which was generated by plotting the true positive rate (sensitivity) against the false positive rate (1—specificity) at various threshold levels. To estimate the confidence interval for the AUC, we employed the DeLong method, which is a non-parametric approach known for providing statistically valid confidence intervals for AUC estimates. Multivariate linear regression modeling was used to reduce type I errors and assess the difference between the patient subgroups (FCF+ versus FCF−) on each of the instruments simultaneously. We standardized variables by centering and scaling them. Specifically, we transformed each variable to have a mean of 0 and a standard deviation of 1. This allows for direct comparisons between the differing metrics of the various survey tools. Data are also shown unscaled for easier interpretation. This study was approved by the University Health Network Research Ethics Board (REB number 23-5563.0), and informed written consent was obtained from each of the participants.

## 3. Results

### 3.1. Study Population

We approached 84 patients for study inclusion and 5 declined study participation. Therefore, we prospectively enrolled 79 individuals, 54 with a Fontan circulation and 25 with a simple shunt lesion, according to predefined inclusion criteria. Following enrollment, no patients were excluded. Baseline demographic and clinical characteristics of the study population are shown (Table 2). As compared with the simple shunt population, those in the Fontan group were less likely to be married/live with a partner, have a university/college degree, or have full-time employment.

The Fontan population was further stratified according to the presence (FCF+) or absence (FCF−) of FCF (Table 3). There were no apparent differences based on age at study entry or sex. However, FCF+ patients were older at the time of Fontan completion, had lower BMI, lower peak VO_2_ on exercise testing, and higher N-terminal pro-brain natriuretic peptide (NT-proBNP) levels. With respect to medical management, FCF+ patients were more likely to be anti-coagulated and to be managed with diuretics as compared with FCF− patients. There were no significant differences in the type of Fontan surgery, the prevalence of arrhythmias (68% versus 48%), or the number of patients with single systemic right ventricle morphology (31% versus 36%) between the Fontan subgroups.

### 3.2. PRO Characteristics According to Subgroup

The PRO measures for each subgroup are shown (Table 4), comparing Fontan versus simple shunts and FCF+ versus FCF− patients. A comparison of FCF+ versus simple shunts and FCF− versus simple shunts, respectively, is shown in the Supplementary Materials Section (Supplementary Table S1). The median KCCQ-12 summary score was lower in the Fontan group compared with the simple shunt group (87 [IQR 79, 95] vs. 100 [IQR 97, 100], *p*-value < 0.001). When the Fontan group was stratified by FCF, the KCCQ-12 score was lower in the FCF+ versus the FCF− group (82 [IQR 56, 89] vs. 93 [IQR 81, 98], *p*-value = 0.002) (see Graphical Abstract). In addition to the KCCQ-12, other PRO measures with statistically different values in the FCF+ subgroup included the EQ5D3L-VAS and the SAS class (Table 4).

### 3.3. KCCQ-12 Discrimination between FCF+ and FCF− Groups

**Figure 1:**
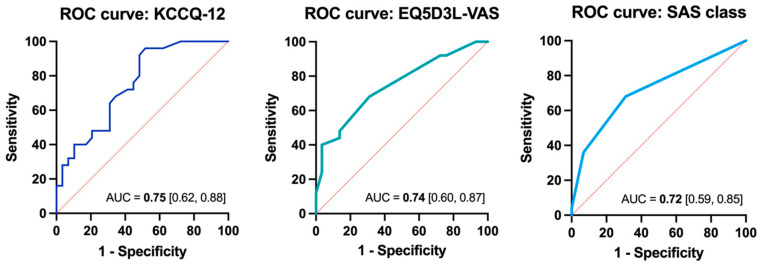
ROC with AUC for KCCQ-12, EQ5D-3L-VAS, and SAS class. AUC, Area Under the Curve; EQ-5D-3L. EuroQol-5 Dimension 3 level version; KCCQ, Kansas City Cardiomyopathy Questionnaire; ROC, Receiver Operating Characteristic; SAS, Specific Activity Scale; VAS, Visual Analogue Scale.

The discriminatory capacity for identification of the FCF+ subgroup was evaluated for each survey tool, using the concordance index. The KCCQ-12 tool demonstrated good discriminatory capacity (c-statistic 0.75 [CI 0.62, 0.88]) along with the EQ5D-3L-VAS and the SAS class (c-statistic 0.74 [CI 0.6–0.87] and 0.72 [CI 0.59–0.85], respectively) (Figure 1 and Supplementary Table S2). The c-statistic for each of the KCCQ-12 subdomains was also calculated (Table S3). The SF-12v2 and the self-reported NYHA class had less robust discrimination for FCF (c-statistic 0.60 [CI 0.44, 0.76] and 0.60 [CI 0.45, 0.74], respectively) (Supplementary Table S2).

The ROC with AUC for survey tools individually and in combination are shown (Table 5). Taken as individual survey tools, the KCCQ-12 and EQ5D-3L-VAS demonstrated very good performance, with robust ROC AUC values and relatively high specificity for FCF identification (79% and 86%, respectively). The SAS class revealed reasonable although slightly lower discrimination for FCF based on the ROC AUC along with somewhat lower specificity (Figure 1). Combining the KCCQ-12 with EQ5D-3L-VAS or the KCCQ-12 with the SAS classification did not result in a substantial change in ROC AUC (Table 5). The highest ROC AUC was observed with the combination of KCCQ-12 along with all other survey tools (ROC AUC 0.82 [0.70, 0.93], sensitivity 68%, specificity 83%).

### 3.4. Differences in PROs According to the Presence of FCF

The multivariable models demonstrate a meaningful difference between FCF+ and FCF− groups in terms of their scores as determined by KCCQ-12, EQ5D-VAS, and SAS class tools, respectively, with similar linear scaled coefficient estimates for each instrument (scaling was used to allow for direct comparisons between differing survey tools) (Table 6). Specific estimates were −0.81 (95% CI −1.3, −0.31; *p* < 0.001) for KCCQ-12, 0.84 (95% CI 0.33, 1.3; *p* < 0.001) for SAS class, and −0.88 (95% CI −1.4, −0.39; *p* < 0.001) for EQ5D-VAS. Models incorporating the mean unscaled differences in values for FCF+ versus FCF− categories for each individual survey tool were also created to allow for a deeper appreciation of the individual characteristics of each instrument (Table 6).

## 4. Discussion

In this study, we examined various PRO measurements in ambulatory Fontan patients with a focus on identifying and characterizing those with FCF. Several novel observations have emerged, namely that (1) HRQOL measures were lower in Fontan patients as compared with patients with simple shunt lesions and the lowest measures were observed in those with FCF; (2) the KCCQ-12 tool allows for good discrimination of FCF; (3) the best discrimination for FCF occurred with the incorporation of multiple complementary PRO tools for assessment of HRQOL in addition to the KCCQ-12 survey.

### 4.1. HRQOL Measures Are Generally Favorable in Adults with a Fontan Circulation

Our study results suggest that overall, self-reported HRQOL measures are relatively favorable in ambulatory patients living with a Fontan circulation. In fact, the EQ5D-VAS median score for patients with a Fontan circulation did not differ significantly as compared with the score for patients with a simple shunt lesion (75 [IQR 60, 80]) versus 80 [IQR 70, 85], *p* = 0.12). In contrast, the KCCQ-12 summary score revealed significant differences in those with a Fontan circulation versus a simple shunt (87 [IQR 76, 95] versus 100 [IQR 97, 100], *p* < 0.001). This observation underscores the importance of appropriate survey tool selection to allow for the identification of important distinguishing features between ACHD patient populations. With this in mind, it is not surprising that some researchers have observed “good” or “excellent” HRQOL in their Fontan cohort [13] while others have not [11,14,20] which may be be attributed to differences in survey tools applied across varying studies. Few studies have explored HF-specific PROs in the Fontan population, and, to date, there have been no published studies specifically focused on PROs in patients within the Fontan subset with FCF.

Beyond variations in selection of the best-suited PRO tool for a given ACHD patient population, conflicting results pertaining to HRQOL measures in the published literature may reflect the inherent heterogeneity of the Fontan population with respect to the type of underlying anatomic lesion, approach to palliation, and hemodynamic sequelae [12,13,14,20]. As expected, measures of HRQOL pertaining to physical limitations were consistently lower in the Fontan as compared with the simple shunt group, as shown in the EQ5D-3L usual activity scale, the self-reported NYHA classification and the SAS score (Table 4). Although differences in physical health differ according to lesion severity, this was not apparent in the domain of mental health. It is notable that almost half of the patients in the simple shunt group identified some degree of difficulty with anxiety/depression based on the EQ5D-3L tool, with findings similar to the Fontan group (44% versus 57%, *p* = 0.3). This observation underscores the mental health disease burden across the spectrum of ACHD lesions, as previously reported [21,22].

### 4.2. Characteristics of Patients with FCF

The KCCQ-12 scores of those admitted with decompensated HF have recently been described in a small cohort of heterogeneous ACHD patients (n = 26 patients) which included only a small number with a Fontan circulation (n = 4); in this study, KCCQ-12 scores improved from admission to discharge [23]. In our study, we observed worse HRQOL in patients with FCF compared to their non-failing counterparts, suggesting that PROs can contribute to the identification of Fontan patients with failing physiology. We observed KCCQ-12 median scores of 82 (IQR 56, 89) in the FCF+ group as compared with 93 (IQR 81, 98) in the FCF− group, which is in keeping with the range of previously published scores in the acquired HF population [6]. The EQ5D-3L questionnaire demonstrated that FCF+ patients had a higher proportion of difficulties in the “usual activities” domain (comprising activities such as work, study, housework, family life, and/or leisure) as compared with the FCF− subgroup (44% versus 17%, *p* = 0.033). Problems with pain/discomfort were apparent with similar proportions in the FCF+ and FCF− subgroups (48% versus 31%, *p* = 0.14), which is in keeping with observations in other ACHD populations by our group and by others [10,24]. Identification and treatment of pain/discomfort may represent an area of focus and potential intervention for ACHD practitioners. Clearly, FCF can have important implications on the life experience of an individual, and future research should be focused on where and how efforts can be directed to enhance support of this vulnerable population.

### 4.3. Identification of Patients with FCF

FCF is the leading cause of morbidity and mortality late after Fontan palliation; however, recognizing early features of FCF can be nuanced and often requires a high level of clinical acumen and expertise. While in centers with many Fontan patients, it is perhaps easier for clinicians to identify failing physiology and structure care for patients to specifically address their needs (e.g., ACHD-HF or Fontan-focused clinics), the value of these survey tools could perhaps be greater for practitioners who follow relatively few Fontan patients, to identify those at risk for earlier referral onwards to more specialized care.

One of the main aims of this study was to reliably identify patients with a vulnerable FCF phenotype and, given the myriad of cardiac and extracardiac manifestations seen in these patients, it is noteworthy that the KCCQ-12, an HF-specific questionnaire, was found to have good discriminatory capacity for identification of these patients. While the KCCQ-12 survey tool alone was a robust discriminator of FCF, it is not surprising that the discriminatory capacity improved when using a combination of different HRQOL survey tools.

### 4.4. Implications for Clinical Practice

Although PRO metrics are increasingly gaining traction for the evaluation of clinical deterioration in patients with acquired HF [25,26], the feasibility of applying PROs to assess HRQOL in specific, high-risk ACHD populations has not previously been described. To our knowledge, this is the first study that reports their use in patients with FCF. Our study identifies three promising survey tools, each with its own relative strength. Compared to other PRO tools, the KCCQ-12 affords the greatest detail, characterizing an array of domains (physical limitations, symptom frequency, social limitations, and quality of life) in addition to providing a summary score, achievable in approximately 2–4 min for completion per participant per visit [27]. The SAS classification, which is a self-reported score corresponding to the conventional physician-assigned New York Heart Association functional classification, allows for a reproducible and reliable assessment of functional capacity based on standardized physical activities. Finally, the EQ5D-VAS, the simplest and quickest of all tools displayed performance characteristics similar to the nuanced KCCQ-12, and as such may be considered a potential screening tool in the Fontan population.

Our study demonstrated the feasibility of using PROs in an ambulatory Fontan population as well as the utility of PRO metrics in identifying those with failing physiology, and may therefore be considered hypothesis-generating. Future directions for study should focus on the longitudinal collection of PRO measures over time with a focus on the predictive value of changes over time with a view to enhanced risk prediction in the Fontan population.

### 4.5. Study Limitations

This was as an exploratory cross-sectional study with inherent limitations by design. These include limited study power related to modest patient numbers and restricted generalizability given results obtained from a single center only. It is worth noting that apparent differences in demographic characteristics between the groups studied may have impacted the interpretation of our results. Future studies with larger numbers from multiple centers could explore the impact of sex or age on PROs in the Fontan population.

Because this study was focused on capturing baseline measures, serial changes in HRQOL were not evaluated and the relationship between PRO tools and clinical worsening over time could not be established. An important research avenue will undoubtedly include further study of the role of PROs for risk prediction in those with a Fontan circulation. Finally, the diversity in our sample may reflect a variety of factors which were beyond the scope of our study (such as access to care, effective transition from pediatric to adult care, etc.).

## 5. Conclusions

The KCCQ-12 questionnaire appears to be a promising tool, either alone or in combination with other HRQOL survey tools, for the identification of patients with FCF. Further research will be needed to establish the value of PRO tools in ACHD clinical practice with a focus on how these can be used to guide therapeutic decisions and/or predict longer-term outcomes in the complex and expanding population of Fontan survivors.

## Figures and Tables

**Table 1:** Health-related quality of life (HRQOL) tools used to evaluate patient reported outcomes (PROs).

PRO	Description
**Kansas City Cardiomyopathy Questionnaire (KCCQ)** [6]	The larger 23-item questionnaire is used to independently measure patients’ perceptions of their health status, including heart failure symptoms, impact on physical and social function, and impact of heart failure on QOL. It includes 6 health state domains and two summary scores: a clinical summary score to correspond with the New York Heart Association (NYHA functional class) and a global summary score incorporating both social and QOL scores.A shorter version with a 12-item questionnaire, the KCCQ-12, provides clinical and overall summary scores with excellent agreement with the full KCCQ tool and was therefore used preferentially in this study [7]. There are 4 domains that measure perception of health status (physical limitations, symptom frequency, QOL and social limitations) as well as a summary score.
**EuroQol-5 Dimension (EQ-5D)** [15]	Five single-item health state dimensions in which patients report degree of limitations by domain (mobility, self-care, usual activities, pain/discomfort, and anxiety/depression) 3 level version (3L) in addition to a Visual Analogue Scale (VAS) that assesses present-day health status and ranges from 0 (“worst imaginable”) to 100 (“best imaginable”) state of health.
**Short Form Health Status Survey (SF-12v2)** [16]	Twelve-item measure with 8 subscales and 2 summary scores with a physical and mental component summary. Standard scores are presented on a 0–100 scale. Higher scores reflect better health status.
**Self-reported New York Heart Association (NYHA) functional class** [17]	Single-item patient reported assessment of NYHA functional class validated in patients with CHD. Worse functional status is reflected in higher classes (from I to IV).
**Specific Activity Scale (SAS)** [18]	Five categories of items exploring ability to perform physical activities (walking, carrying items, household activities, and self-care) are combined to assign a patient to a functional class (from 1 to IV) corresponding to the NYHA classification. Worse functional status is reflected in higher classes (from I to IV).

**Table 2:** Baseline demographic and characteristics of the study population.

	Fontan (n = 54)	Simple Shunts (n = 25)	*p*-Value
Demographic data			
Age	34 (29, 41)	32 (27, 43)	0.740
Male	34 (63%)	10 (40%)	0.080
White	42 (78%)	14 (56%)	0.131
BMI	25.2 (21.4, 29.1)	24.5 (22.2, 28.7)	0.887
Married/living with a partner	22 (41%)	17 (68%)	**0.024**
Parent (patient has children)	12 (22%)	10 (40%)	0.112
College or university degree	28 (52%)	19 (76%)	**0.042**
Learning disability (self-reported)	20 (37%)	4 (16%)	0.052
Employed (full or part time)	36 (67%)	22 (88%)	**0.023**
Receiving governmental financial aid	9 (17%)	0 (0%)	0.051
Cardiovascular risk factors			
Systemic hypertension	1 (2%)	2 (8%)	0.234
Smoking history	6 (11%)	1 (4%)	0.422
Known CAD	1 (2%)	2 (8%)	0.234
History of stroke	6 (11%)	0 (0%)	0.169
Diabetes Mellitus	3 (6%)	0 (0%)	0.548
Obesity (BMI > 30 kg/m^2^)	12 (22%)	3 (12%)	0.365

BMI, Body Mass Index; CAD, Coronary Artery Disease.

**Table 3:** Baseline demographic and medical characteristics of patients in the Fontan group stratified by Fontan circulatory failure (FCF).

	FCF+ (n = 25)	FCF− (n = 29)	*p*-Value
Demographic Data			
Age	38 (29, 46)	33 (29, 36)	0.081
Male	13 (52%)	21 (72%)	0.197
White	22 (88%)	20 (69%)	0.476
BMI	22.6 (20.8, 26.0)	26.0 (23.4, 29.4)	**0.048**
Married/Living with a partner	8 (32%)	14 (48%)	0.225
Parent (patient has children)	4 (16%)	8 (28%)	0.344
College or university degree	9 (36%)	19 (66%)	**0.03** **0**
Self-reported learning disability	12 (48%)	8 (28%)	0.094
Employed (full or part time)	24 (96%)	12 (41%)	**0.015**
Receiving governmental financial aid	2 (8%)	7 (24%)	0.065
Cardiac anatomy			
Tricuspid atresia	6 (24%)	8 (28%)	0.764
Pulmonary atresia with VSD	0 (0%)	1 (3%)	1.000
Pulmonary atresia with IVS	2 (8%)	2 (7%)	1.000
DORV	3 (12%)	3 (10%)	1.000
HLHS	5 (20%)	3 (10%)	0.499
Unbalanced AVSD	2 (8%)	3 (10%)	1.000
DILV	4 (16%)	5 (17%)	1.000
Straddling AV valve	3 (12%)	4 (14%)	1.000
Cardiovascular risk factors			
Smoking history	5 (20%)	1 (3%)	0.085
Systemic hypertension	0 (0%)	1 (3%)	1.000
Known CAD	1 (4%)	0 (0%)	0.463
History of stroke	3 (12%)	3 (10%)	1.000
Diabetes Mellitus	2 (8%)	1 (3%)	0.591
Obesity (BMI > 30 kg/m^2^)	5 (20%)	7 (24%)	0.715
Fontan characteristics			
Age at Fontan completion	6 (4, 13)	3 (2, 5)	**0.003**
Type of Fontan surgery			0.802
Atriopulmonary connection	7 (28%)	6 (21%)	
Lateral	6 (24%)	8 (28%)	
Extracardiac	10 (40%)	14 (48%)	
Other	2 (8%)	1 (3%)	
Systemic RV	9 (36%)	9 (31%)	0.700
Systemic LV	16 (64%)	20 (69%)	0.700
Chronic cyanosis (O_2_ saturation <90%)	15 (60%)	10 (34%)	0.061
History of atrial arrhythmia	17 (68%)	14 (48%)	0.144
Hospital admissions (last year) *	14 (56%)	1 (3%)	**<0.001**
NT-proBNP	538 (355, 842)	192 (66, 298)	**<0.001**
Echocardiography ^#^			
Degree of AV valve regurgitation			0.884
None/Trivial	7 (28%)	9 (31%)	
Mild	6 (24%)	9 (31%)	
Moderate	9 (36%)	9 (31%)	
Severe	3 (12%)	2 (7%)	
Degree of aortic valve regurgitation			0.136
None/Trivial	12 (48%)	17 (59%)	
Mild	6 (24%)	10 (34%)	
Moderate	7 (28%)	2 (7%)	
Severe	0 (0%)	0 (0%)	
Systemic EF			0.281
Normal (≥53%)	14 (56%)	23 (79%)	
Mild (41 to 52%)	3 (12%)	1 (3%)	
Moderate (30 to 40%)	5 (20%)	4 (14%)	
Severe (<30%)	3 (12%)	1 (3%)	
ECG			
Rhythm			0.196
Sinus	14 (56%)	23 (79%)	
Paced	5 (20%)	4 (14%)	
A-tach	2 (8%)	0 (0%)	
Other	4 (16%)	2 (7%)	
CPET			
Peak VO_2_ (mL/kg/min)	18.5 (6.5)	22.0 (4.0)	**0.006**
Peak VO_2_ (% predicted)	55 (17)	60 (11)	0.081
VO_2_ at AT (mL/kg/min)	11.8 (3.9)	14.6 (2.3)	**0.003**
VO_2_ at AT (% predicted)	37 (11)	39 (7)	0.393
Lowest O_2_ saturation during exercise	87.9 (5.6)	90.0 (5.1)	0.222
Medical therapies			
Diuretic	18 (72%)	4 (14%)	**<0.001**
Beta blockade	15 (60%)	12 (41%)	0.172
ACEi/ARB/ARNI	3 (12%)	8 (28%)	0.156
MRA	16 (64%)	3 (10%)	**<0.001**
SGLT2i	3 (12%)	1 (3%)	0.326
ASA	8 (32%)	12 (41%)	0.477
Anti-coagulation	19 (76%)	14 (48%)	**0.037**

* At least one admission within the last year for arrhythmia or heart failure; ^#^ Aortic and systemic AV valve regurgitation were graded as normal if there was no valvular regurgitation, and mild, moderate, or severe according to criteria provided by the American Society of Echocardiography (ASE) Guidelines [19]. ASA, Acetylsalicylic Acid; AT, Anaerobic Threshold; ACEi, Angiotensin-converting Enzyme Inhibitors; ARB, Angiotensin Receptor Blocker; ARNI, Angiotensin Receptor/Neprilysin Inhibitor; A-tach, atrial tachycardia; AV, atrioventricular; AVSD, Atrioventricular Septal Defect; BMI, Body Mass Index; CPET, cardiopulmonary exercise testing; CAD, Coronary Artery Disease; EF, Ejection Fraction; ECG, electrocardiogram; FCF, Fontan circulatory failure; DILV, Double Inlet Left Ventricle; DORV, Double Outlet Right Ventricle; HLHS, Hypoplastic Left Heart Syndrome; IVS, Intact Ventricular Septum; LV, Left Ventricle; MRA, Mineralocorticoid Receptor Antagonist; NT-proBNP, N-terminal Prohormone of Brain Natriuretic Peptide; n, number; O_2_, oxygen; VO_2_, oxygen consumption; RV, right ventricle; SLGT2i, Sodium–Glucose Cotransporter-2 Inhibitors; VSD, ventricular septal defect.

**Table 4:** Patient reported outcome (PRO) characteristics according to subgroup (Fontan versus simple shunt and FCF+ versus FCF−).

PRO Measure	Fontan (n = 54)	Simple Shunt (n = 25)	*p*-Value	FCF+ (n = 25)	FCF– (n = 29)	*p*-Value
**KCCQ-12 summary score**	87 (76, 95)	100 (97, 100)	**<0.001**	82 (56, 89)	93 (81, 98)	**0.002**
**KCCQ-12 domains**						
Physical limitations	83 (69, 92)	100 (92, 100)	**<0.001**	83 (67, 92)	92 (75, 100)	0.061
Symptom frequency	88 (71, 100)	100 (96, 100)	**<0.001**	75 (65, 92)	100 (85, 100)	**0.002**
Social limitations	92 (77, 100)	100 (100, 100)	**<0.001**	83 (67, 100)	100 (92, 100)	**0.011**
Quality of life	88 (62, 88)	100 (88, 100)	**<0.001**	75 (38, 88)	88 (75, 100)	**0.002**
**EQ-5D VAS**	75 (60, 80)	80 (70, 85)	0.122	70 (50, 75)	75 (70, 85)	**0.002**
**EQ-5D-3L**	83 (67, 100)	100 (83, 100)	**0.024**	75 (59, 84)	83 (78, 100)	**0.033**
**EQ-5D problems**						
Mobility	10 (19%)	2 (8%)	0.320	7 (28%)	3 (10%)	0.102
Self-care	5 (9%)	0 (0%)	0.173	3 (12%)	2 (7%)	0.534
Usual activity	16 (30%)	2 (8%)	**0.033**	11 (44%)	5 (17%)	**0.034**
Pain/discomfort	21 (39%)	5 (20%)	0.097	12 (48%)	9 (31%)	0.210
Anxiety/depression	31 (57%)	11 (44%)	0.267	17 (68%)	14 (48%)	0.151
**SF-12v2**						
PCS	38.7 (34, 42)	39.7 (38, 42)	0.081	38.9 (38, 42)	37.2 (34, 41)	0.379
MCS	48 (42, 53)	46 (43, 51)	0.330	47 (39, 51)	49 (46, 53)	0.317
**NYHA class**			**<0.001**			0.194
1	20 (37%)	21 (84%)		8 (32%)	12 (41%)	
2	21 (39%)	4 (16%)		9 (36%)	12 (41%)	
3	9 (17%)	0 (0%)		4 (16%)	5 (18%)	
4	4 (7%)	0 (0%)		4 (16%)	0 (0%)	
**SAS class**			**0.027**			**0.015**
1	28 (52%)	20 (80%)		8 (32%)	20 (69%)	
2	15 (28%)	5 (20%)		8 (32%)	7 (24%)	
3	10 (18%)	0 (0%)		8 (32%)	2 (7%)	
4	1 (2%)	0 (0%)		1 (4%)	0 (0%)	

EQ-5D-3L, EuroQol-5 Dimension 3 level version; FCF, Fontan circulatory failure; KCCQ-12, 12-item shorter version of the Kansas City Cardiomyopathy Questionnaire; MCS, Mental Component Score; NYHA, New York Heart Association; PRO, patient reported outcome; PCS, Physical Component Score; SF-12v2, Short form Health Status Survey Version 2; SAS, Specific Activity Scale; VAS, Visual Analogue Scale.

**Table 5:** Survey tool metrics are shown individually and in combination.

Instrument	ROC AUC (95% CI)	Sensitivity	Specificity	Accuracy
**Individual surveys**				
KCCQ12	0.75 (0.62, 0.88)	0.44	0.79	0.63
EQ5D-VAS *	0.74 (0.60, 0.87)	0.48	0.86	0.69
SF-12v2	0.60 (0.44, 0.76)	0.40	0.79	0.61
SAS class	0.72 (0.59, 0.85)	0.68	0.69	0.69
NYHA class	0.60 (0.45, 0.74)	0.32	0.83	0.59
**KCCQ-12 + one additional survey**				
KCCQ12 + EQ5D-3L-VAS	0.77 (0.64, 0.90)	0.44	0.90	0.69
KCCQ12 + SF-12v2	0.75 (0.62, 0.88)	0.44	0.83	0.65
KCCQ12 + SAS class	0.77 (0.64, 0.89)	0.44	0.83	0.65
KCCQ12 + NYHA class	0.77 (0.64, 0.90)	0.48	0.83	0.67
**Incremental value of KCCQ12 + multiple additional surveys**				
KCCQ12 + EQ5D-3L-VAS + SAS class	0.77 (0.64, 0.90)	0.44	0.83	0.69
KCCQ12 + EQ5D-3L-VAS + SAS class + SF-12v2	0.79 (0.68, 0.91)	0.48	0.79	0.65
KCCQ12 + EQ5D-3L-VAS + SAS class + SF-12v2 + NYHA class	0.82 (0.70, 0.93)	0.68	0.83	0.76

* as compared with the full EQ5D-3L-VAS survey tool, when the EQ5D-VAS was studied alone, the ROC AUC was unchanged at 0.74 with sensitivity 0.48 and specificity 0.86. AUC, Area Under the Curve; CI, confidence interval; EQ-5D, EuroQol-5 Dimension; KCCQ, Kansas City Cardiomyopathy Questionnaire; MCS, Mental Component Score; NYHA, New York Heart Association; PCS, Physical Component Score; ROC, Receiver Operating Characteristic; SAS, Specific Activity Scale; SF-12v2, Short form Health Status Survey Version 12; VAS, Visual Analogue Scale.

**Table 6:** Multivariable models.

		Scaled (Mean Difference)		Unscaled (Mean Difference)		
Instrument	Class	Estimate	95% CI	Estimate	95% CI	*p*-Value
Reference category	FCF−					
KCCQ-12	FCF+	−0.81	−1.3, −0.31	−15.29	−25, −5.8	**0.002**
EQ-5D-3L	FCF+	−0.64	−1.2, −0.11	−12.18	−22, −2.1	**0.018**
EQ-5D-VAS	FCF+	−0.88	−1.4, −0.39	−16.22	−25, −7.1	**0.001**
SAS Class	FCF+	0.84	0.33, 1.3	0.7	0.28, 1.1	**0.002**
NYHA Class	FCF+	0.44	−0.10, 0.98	0.4	−0.09, 0.9	0.111
SF12v2-MCS	FCF+	−0.31	−0.86, 0.23	−2.66	−7.3, 2	0.254
SF12v2-PCS	FCF+	0.30	−0.24, 0.85	1.65	−1.3, 4.6	0.272

CI, confidence interval; EQ-5D-3L, EuroQol-5 Dimension 3 level version; FCF, Fontan circulatory failure; KCCQ, Kansas City Cardiomyopathy Questionnaire; MCS, Mental Component Score; NYHA, New York Heart Association; PCS, Physical Component Score; SAS, Specific Activity Scale; VAS, Visual Analogue Scale.

## Data Availability

The original contributions presented in the study are included in the article and supplementary material; further inquiries can be directed to the corresponding author.

## Supplementary Materials

The following supporting information can be downloaded at https://www.mdpi.com/article/10.3390/jcm13144175/s1, Table S1: Patient reported outcome (PRO) characteristics according to subgroup (simple shunts vs. FCF− and simple shunts vs. FCF+); Table S2: Concordance indices for PRO tools compared to KCCQ12 as the reference tool; Table S3: Concordance index of the KCCQ-12 stratified by survey domain.
