# Supplementary material for: Patient Reported Outcome Measures in Adults with Fontan Circulatory Failure
Source: J Clin Med. 2024 Jul 17;13(14):4175. doi: 10.3390/jcm13144175 (PMC11277569; doi:10.3390/jcm13144175)
Supplement: Supplementary file 1 [file jcm-13-04175-s001.zip › jcm-3062939-supplementary update.pdf]

SUPPLEMENTARY MATERIALS

Table S1. Patient report outcome (PRO) characteristics according to subgroup (Simple Shunts vs FCF- and Simple Shunts vs FCF+).

**PRO measure**	**FCF-** (n= 29)	**Simple shunt** (n = 25)	**p-value**	**FCF+** (n= 25)	**Simple shunt** (n = 25)	**p-value**
**KCCQ-12 summary score**	93 [81, 98]	100 [97, 100]	**0.002**	82 [56, 89]	100 [97, 100]	**<0.001**
**KCCQ-12 domains**						
Physical limitations	92 [75, 100]	100 [92, 100]	**0.002**	83 [67, 92]	100 [92, 100]	**<0.001**
Symptom frequency	100 [71, 100	100 [96, 100]	0.176	75 [65, 92]	100 [96, 100]	**<0.001**
Social limitations	92 [77, 100]	100 [100, 100]	**0.005**	83 [67, 100]	100 [100, 100]	**<0.001**
Quality of life	88 [75, 100]	100 [88, 100]	**0.007**	75 [38, 88]	100 [88, 100]	**<0.001**
**EQ-5D VAS**	75 [70, 85]	80 [70, 85]	1	70 [50, 75]	80 [70, 85]	**0.004**
**EQ-5D-3L**	83 [78, 100]	100 [83, 100]	0.282	75 [59, 84]	100 [83, 100]	**0.003**
**EQ-5D problems**						
Mobility	3 (10%)	2 (8%)	1	7 (28%)	2 (8%)	0.138
Self-care	2 (6.9%)	0 (0%)	0.493	3 (12%)	0 (0%)	0.235
Usual activity	5 (17%)	2 (8%)	0.431	11 (44%)	2 (8%)	**0.004**
Pain/discomfort	9 (31%)	5 (20%)	0.356	12 (48%)	5 (20%)	**0.037**
Anxiety/depression	14 (48%)	11 (44%)	0.753	17 (68%)	11 (44%)	0.087
**SF-12v2**						
PCS	37.2 [34, 41]	39.7 [38, 42]	**0.043**	38.9 [38, 42]	39.7 [38, 42]	0.347
MCS	48.6 [46, 53]	46 [43, 51]	0.101	47 [39, 51]	46 [43, 51]	0.977
**NYHA class**			**0.003**			**0.001**
1	12 (41%)	21 (84%)		8 (32%)	21 (84%)	
2	12 (41%)	4 (16%)		9 (36%)	4 (16%)	
3	5 (18%)	0 (0%)		4 (16%)	0 (0%)	
4	4 (7.4%)	0 (0%)		4 (16%)	0 (0%)	
**SAS class**			0.515			**0.001**
1	20 (69%)	20 (80%)		8 (32%)	20 (69%)	
2	7 (24%)	5 (20%)		8 (32%)	5 (20%)	
3	2 (7%)	0 (0%)		8 (32%)	0 (0 %)	
4	0 (0%)	0 (0%)		1 (4.0%)	0 (0%)	

EQ-5D-3L, EuroQol-5 Dimension 3 level version; EQ-5D, EuroQol-5 Dimension; FCF, Fontan Circulatory Failure; KCCQ-12, 12-item shorter version of the Kansas City Cardiomyopathy Questionnaire; MCS, Mental Component Score; NYHA, New York Heart Association; PRO, Patient Reported Outcomes; PCS, Physical Component Score; SF-12v2, Short Form Health Status Survey Version 2; SAS, Specific Activity Scale; VAS, Visual Analogue Scale.

Table S2. Concordance indices for PRO tools compared to KCCQ12 as the reference tool.

PRO tool	C-statistic (CI)	p-value*
KCCQ	0.75 (0.62, 0.88)	--
EQ5D-3L-VAS	0.74 (0.6, 0.87)	0.850
SAS class	0.72 (0.59, 0.85)	0.625
SF12v2	0.6 (0.44, 0.76)	0.113
MCS	0.58 (0.44, 0.76)	0.068
PCS	0.57 (0.41, 0.73)	0.086
NYHA class	0.6 (0.45, 0.74)	0.005

*p-values reflect the comparison between the individual survey tool and the KCCQ-12 survey. CI, Confidence Interval; EQ-5D, EuroQol-5 Dimension; KCCQ, Kansas City Cardiomyopathy Questionnaire; MCS, Mental Component Score; NYHA, New York Heart Association; SF-12v2, PCS, Physical Component Score; Short Form Health Status Survey Version 2; SAS, Specific Activity Scale; VAS, Visual Analogue Scale.

Table S3. Concordance index of the KCCQ-12 stratified by survey domain.

Characteristics	C-statistic	Lower CI	Upper CI
KCCQ (Summary score)	0.75	0.62	0.88
KCCQ (Symptom frequency)	0.74	0.61	0.86
KCCQ (Social limitations)	0.69	0.61	0.86
KCCQ (Quality of life)	0.74	0.55	0.83
KCCQ (Physical limitations)	0.65	0.50	0.79

CI, Confidence Interval; KCCQ, Kansas City Cardiomyopathy Questionnaire.
